# A Portable RT-LAMP/CRISPR Machine for Rapid COVID-19 Screening

**DOI:** 10.3390/bios11100369

**Published:** 2021-10-02

**Authors:** Meysam Rezaei, Sajad Razavi Bazaz, Dorsa Morshedi Rad, Olga Shimoni, Dayong Jin, William Rawlinson, Majid Ebrahimi Warkiani

**Affiliations:** 1 School of Biomedical Engineering, University of Technology Sydney, Sydney, NSW 2007, Australia; meysam.rezaei@genea.com.au (M.R.); Sajad.RazaviBazaz@student.uts.edu.au (S.R.B.); Dorsa.MorshediRad@student.uts.edu.au (D.M.R.); 2 Institute for Biomedical Materials & Devices (IBMD), Faculty of Science, University of Technology Sydney, Sydney, NSW 2007, Australia; Olga.Shimoni@uts.edu.au (O.S.); Dayong.Jin@uts.edu.au (D.J.); 3 Genea, Sydney, NSW 2000, Australia; 4 SUStech-UTS Joint Research Centre for Biomedical Materials & Devices, Southern University of Science and Technology, Shenzhen 518055, China; 5 Serology and Virology Division, NSW Health Pathology, Prince of Wales Hospital, Sydney, NSW 2031, Australia; w.rawlinson@unsw.edu.au; 6 School of Women’s and Children’s Health, University of New South Wales, Sydney, NSW 2052, Australia

**Keywords:** SARS-CoV-2, COVID-19, point of care testing, rapid diagnostics

## Abstract

The COVID-19 pandemic has changed people’s lives and has brought society to a sudden standstill, with lockdowns and social distancing as the preferred preventative measures. To lift these measurements and reduce society’s burden, developing an easy-to-use, rapid, and portable system to detect SARS-CoV-2 is mandatory. To this end, we developed a portable and semi-automated device for SARS-CoV-2 detection based on reverse transcription loop-mediated isothermal amplification followed by a CRISPR/Cas12a reaction. The device contains a heater element mounted on a printed circuit board, a cooler fan, a proportional integral derivative controller to control the temperature, and designated areas for 0.2 mL Eppendorf^®^ PCR tubes. Our system has a limit of detection of 35 copies of the virus per microliter, which is significant and has the capability of being used in crisis centers, mobile laboratories, remote locations, or airports to diagnose individuals infected with SARS-CoV-2. We believe the current methodology that we have implemented in this article is beneficial for the early screening of infectious diseases, in which fast screening with high accuracy is necessary.

## 1. Introduction

The SARS-CoV-2 virus causes COVID-19, a serious and life-threatening disease with a wide spectrum of symptoms, ranging from fever and cough to dyspnea, and in severe cases, resulting in intensive care unit (ICU) admission of patients, organ failure, and death [1,2]. The continuing spread of COVID-19 and its high transmissibility have become a major concern and prompted the rapid development of diagnostic methods. Conventional diagnostic methods such as quantitative reverse transcription polymerase chain reaction (RT-qPCR) have been developed and are used routinely for SARS-CoV-2 detection [3]. Although RT-qPCR is the gold standard technique to diagnose SARS-CoV-2, laboratories with RT-qPCR capabilities are often centralized, making this method less suitable in settings such as aged care facilities and ports of entry where on-site screening is required [4]. In addition, other diagnostic methods have been developed based on the detection of antibodies and antigens, which are simple, quick, and cheap and do not necessitate the use of specific instrumentation or trained users [5]. Since antibodies against SARS-CoV-2 take days to weeks to be produced in the patient’s body, antigen-based methods are not recommended to diagnose acute COVID-19 cases. Moreover, these diagnostic tests targeting the viral proteins are usually less sensitive than RT-qPCR tests detecting the viral nucleic acids. Therefore, these tests may not be considered highly sensitive methods for early detection of COVID-19 [6].

Recent studies have focused on the development of integrated molecular diagnostic devices based on microfabrication technologies to provide rapid and reliable methods for SARS-CoV-2 detection, enabling faster clinical decisions [7]. In this study, we report the development and validation of a portable, integrated, and semi-automated device for in vitro COVID-19 molecular diagnosis. This device involves nucleic acid reverse transcription and isothermal amplification using loop-mediated amplification (RT-LAMP) of viral ribonucleic acid (RNA) extracted from nasopharyngeal or oropharyngeal swabs followed by Cas12a detection and readout with lateral flow assay (LFA) strips. Given the low reagent consumption, the compact size, and integration capability with a power bank, this wireless device can be conveniently used in crisis centers, mobile laboratories, remote locations, or airports to diagnose individuals infected with SARS-CoV-2. Our device consists of a small thermocycler machine integrated into a portable box containing sample tubes that can be potentially used for simultaneous testing of five tests in just 35 min with minimal hands-on processing.

**Figure 1:**
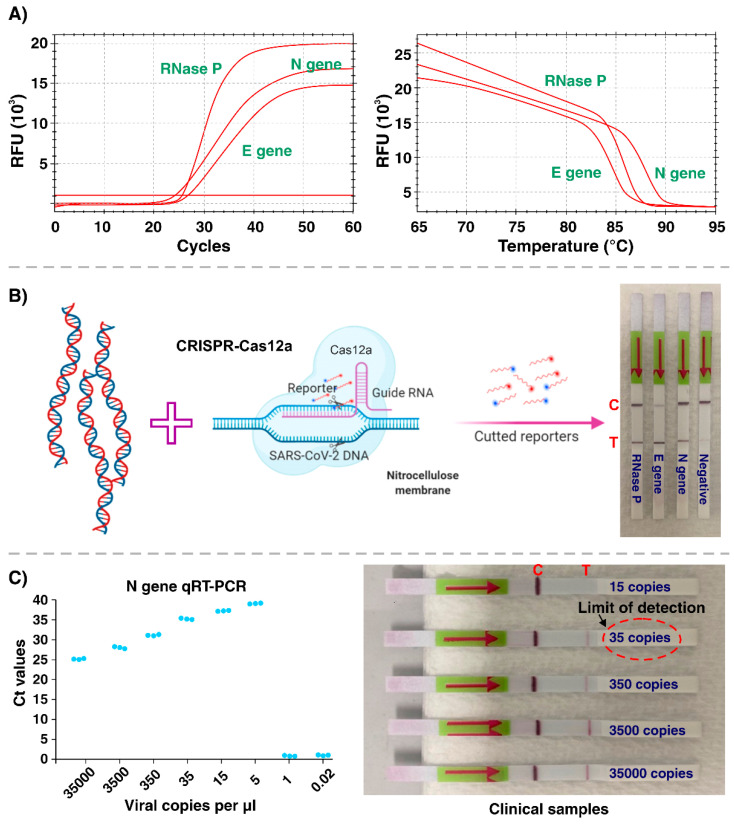
(**A**) RT-LAMP reaction performed in an RT-qPCR machine using N, E, and RNase P primers at an optimal temperature and time. (**B**) Schematic illustration of CRISPR-Cas12a reaction on pre-amplified SARS-CoV-2 target genes, followed by visualization with lateral flow strips. (**C**) The detection limit of the RT-qPCR assay (left) using PCR machine and RT-LAMP/Cas12a assay (right) using clinical samples.

A total of 10 samples were collected from Serology and Virology laboratories at the Prince of Wales Adult Hospital (Sydney, NSW, Australia) under the Biosafety (ETH-5127) protocol. Each patient, who had symptoms of respiratory disease and a positive RT-qPCR result for COVID-19, participated in this study voluntarily after signing an informed consent form. Next, the patient’s nasopharyngeal or oropharyngeal swab sample was collected in the Copan universal transport medium for viruses and vortexed at maximum speed for 1–2 min followed by passing through the 0.45 µm Minisart^®^ filter (Sartorius Stedim Biotech, Goettingen, Germany). Then viral RNA was extracted from 200 µL of samples using the MagNA Pure LC Total Nucleic Acid Isolation Kit (Roche Diagnostics, Sydney, Australia) according to the manufacturer’s instructions. Then, the purity and quantity of extracted RNA samples were evaluated using NanoDrop (One UV-Vis Spectrophotometer, Thermofisher Scientific, Waltham, MA, USA) and stored at −80 °C for further analysis. First, we set the RT-LAMP primers to target the E (envelope) and N (nucleoprotein) genes of SARS-CoV-2, covering the regions followed by protocols validated by the World Health Organization (WHO) and US Centers for Disease Control and Prevention (CDC) assays, as well as the RNase P gene as a control [8]. To this aim, the RT-Lamp reaction for the preamplification of viral RNA has been performed by preparing 25 µL of a reaction mix containing the WarmStart LAMP master mix (2×), fluorescent dye (50×), and 4 µM LAMP primer mix followed by incubation at 65 °C for 30 min [9]. Then, we set the Cas12a gRNAs to detect the control gene, E gene, and specifically the N gene in SARS-CoV-2 [8]. We conducted a series of experiments using a higher concentration of SARS-CoV-2 RNA (1 × 10^5^ copies/µL) to assess the functionality of the RT-LAMP reactions in a q-PCR machine (CFX Connect™ Real-Time PCR System. Bio-Rad, Hercules, CA, USA). Using this q-PCR apparatus and fluorescent intercalating dye in the first place enabled us to track the RT-LAMP reaction in real-time and optimize the optimal temperature and time for SARS-CoV-2 RNA gene target amplification. The amplification was performed at a temperature gradient of 58 to 70 °C for 30 min. The optimal amplification proceeded at 65 °C for 25 min (Figure 1A), and no amplification was observed in negative samples (containing all the reaction materials except viral RNA). We then evaluated the performance of the detection step by combining pre-amplified viral and control RNA targets with the Cas12a-gRNA ribonucleoprotein (RNP) assays followed by the LFA strips readout (Figure 1B). Each pre-amplified target was added to a separate tube containing a specific Cas12a-gRNA and a FAM-biotin reporter molecule, prepared as suggested by the assay manufacturer (New England Biolabs, Ipswich, MA, USA) [9]. After incubation for 10 min at 37 °C, the completed reaction was read out using LFA strips placed into the tubes, showing the results after 2 min.

We observed concordance between our results from fluorescence-based readouts for positive/negative RT-LAMP samples in q-PCR and those from lateral flow strips by the appearance of one (negative sample) or two (positive sample) lines. With the presence of N, E, and Rnase P target genes amplified during the RT-LAMP step, Cas12a can specifically bind to targets using its gRNAs, resulting in the activation of indiscriminate Cas12a cleavage and cutting of the FAM-biotin reporter molecules. Visualization of the Cas12a cleavage activity was achieved by applying LFA strips designed to capture FAM- and Biotin-tagged nucleic acids (Milenia HybriDetect 1, TwistDx, Cambridge, UK). For the positive samples, two lines were observed on the lateral flow strips: Uncut reporters captured at the first line (control) and cut reporters captured at the second line (test); while in negative samples, only one line (control) of captured uncut reporters was detected.

After validating the feasibility of our approach for detecting the SARS-CoV-2 virus, we determined the limit of detection (LoD) in a controlled situation using lab-based instruments before the final design and production of our portable RT-LAMP/CRISPR machine for rapid COVID-19 screening. First, we quantified the number of viral copies in one sample using q-PCR (N = 3) after preparing a standard curve using a GenScript RT-qPCR detection assay. The standard curve was prepared using six dilutions of N-positive control plasmids (3 × 10^5^ copies/µL) with three replicates at each dilution. After determining the RNA copy numbers in our selected sample, we prepared a series of diluted samples with concentrations of 35,000, 3500, 350, 35, 15, 5, 1, and 0.02 copies per µL. The copy number of each sample was confirmed and the LoD of commercial RT-qPCR assay for SARS-CoV-2 detection was determined using the cycle threshold (Ct) values (Figure 1C). Finally, we determined the LoD of the RT-LAMP/Cas12a assay by assessing the ability to generate visible test and control lines on lateral flow strips after testing three replicates of all eight diluted samples. The minimum copy numbers that could be detected using the RT-qPCR assay was 5 copies per µL (consistent in all 3 replicates), compared with 35 copies per µL of targets with the RT-LAMP/CRISPR-Cas12a approach (Figure 1C).

**Figure 2:**
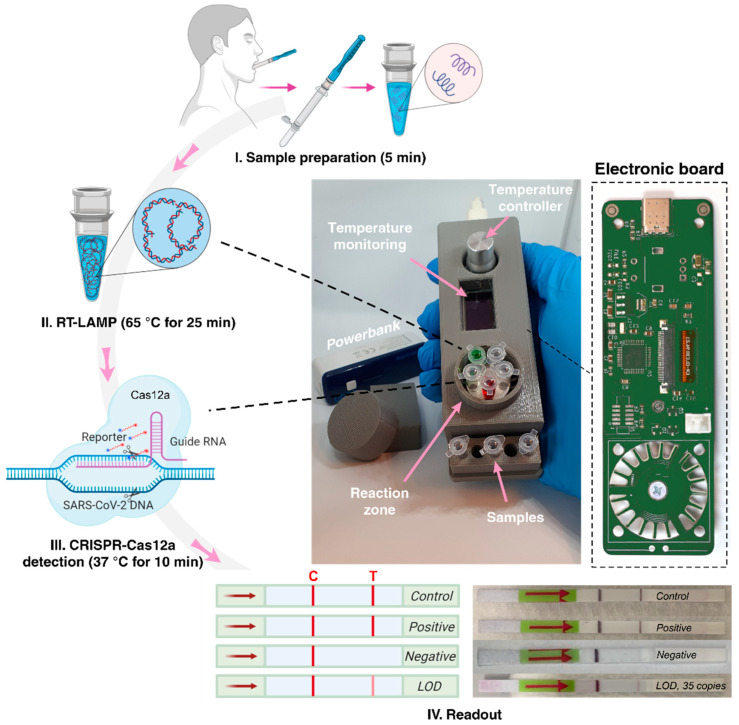
Schematic representation of the experimental procedure for the detection of SARS-CoV-2 with a portable device using an RT-LAMP/CRISPR-Cas12a approach. The workflow includes SARS-CoV-2 virus sampling from confirmed patients, virus deactivation and RNA extraction, RNA transcription and amplification by RT-LAMP, Cas12a reaction, and lateral flow strips visualization. Our portable device, along with its integrated electronic board, is demonstrated at the center of the figure. Samples in red and green are food dye for better illustration.

Having shown the proof of concept of our approach in the laboratory setting, modifications were made to build a portable device to be utilized for SARS-CoV-2 point-of-care testing. The RT-LAMP step requires a heater that should have accurate thermal stability, i.e., the temperature at each step should be accurate and consistent without variation. Although laboratory instruments for thermal control of various tubes and vessels exist, these facilities are often bulky and expensive. Moreover, in some of these facilities, condensation occurs on the tube walls and lid, resulting in performance loss and reduced efficiency [10]. As a result, they are not favorable for point-of-care devices, especially in resource-poor settings. Although there are ongoing efforts to improve lab-scale heaters’ temperature uniformity, this feature is yet to be optimized and is not fully functional [11]. To address this issue and make the process semi-automated, we designed a portable machine for accurate control of the heating process during the experiment. In our design, a heater element is mounted on a printed circuit board (PCB). A small fan that is directly connected to the board was used to reduce the temperature. The device is able to reach any temperatures within the range of 20 to 95 °C. The system’s temperature is controlled and regulated using a proportional integral derivative (PID) controller, which is among the most stable, reliable, and accurate controllers and uses a loop feedback approach to control the system [12]. These components were mounted on a PCB board, programmed using Python and layout software packages. Tubes with reagents were placed on the heater with a closed lid for better temperature control and enhanced efficiency. The device operates on power obtained from a USB Type-C connection, meaning that it can be connected to a USB port of any computer, Power Bank, or battery with a minimum rating of 2A. The device can fit in a single hand or a pocket, with approximate dimensions of 3.5 cm × 3 cm × 13 cm, and can handle up to 10 samples in the sample position and reaction zone (Figure 2). Python programing of the system enabled us to turn on the device at a specific time and increase or decrease the temperature of the system at a specific point. This means that amplification and detection in our system are essentially automated (except for tube handling). Because the PID controller adjusts the temperature, and the reaction zone (Figure 2) is relatively small, there is no heat loss or temperature variation in the system, with minimum condensation on tube lids and walls. To decrease the temperature for the CRISPR/Cas12a reaction, the fan reduces the temperature of the RT-LAMP step to 37 °C directly via the PID controller.

We have repeated SARS-CoV-2 detection for all 10 patient samples using the modified portable device. The device was connected to a 2600 mAh Cellularline Power Bank, which has an estimated battery life of up to 10 h without the need of recharging, representing a significant step forward for a point-of-care diagnostic device. For the RT-LAMP stage, the temperature was set at 65 °C for 25 min. After completing this stage, the temperature was set at 37 °C for 10 min for the CRISPR/Cas12a reaction. To minimize the risk of cross-contamination between the test samples, microtubes are moved and opened in the sample position once the RT-LAMP reaction has been completed. Finally, the LFA strips were placed in the tubes and the results were analyzed. As shown in Figure 2, all the results were replicable, and an LOD of 35 copies was achieved, similar to that obtained using a lab-based PCR machine.

In conclusion, we were able to successfully design and develop a low-cost, portable, and semi-automated device leveraging RT-LAMP/CRISPR technologies that can be used for early SARS-CoV-2 detection with an LOD of 35 copies per microliter. Through multiplexing our portable machine and increasing the number of wells in the reaction zone, up to 500 samples can be processed simultaneously in just 35 min. Moreover, with multiplexing the CRISPR/Cas technology, our device can offer co-detection of SARS-CoV-2 and the flu in a single reaction with minimal sample requirements. After further screening of different SARS-CoV-2 variants in several patient populations, our portable machine holds significant potential for being rapidly used as a point-of-care testing device in crisis centers and mobile laboratories.

## Data Availability

The data presented in this study are available on request from the corresponding author.
